# Multicenter clinical evaluation of a multi-dose formulation of propofol in the dog

**DOI:** 10.1186/1746-6148-9-261

**Published:** 2013-12-23

**Authors:** Khursheed R Mama, James S Gaynor, Ralph C Harvey, Sheilah A Robertson, Robbin L Koenig, Elizabeth M Cozzi

**Affiliations:** 1 Department of Clinical Sciences, College of Veterinary Medicine and Biomedical Sciences, Colorado State University, Fort Collins, CO 80523, USA; 2 Peak Performance Veterinary Group, Colorado Springs, Co 80918, USA; 3 Department of Small Animal Clinical Sciences, College of Veterinary Medicine, University of Tennessee, Knoxville, TN 37996, USA; 4 Department of Large Animal Clinical Sciences, College of Veterinary Medicine, University of Florida, Gainesville, FL 32608, USA; 5 Biotechnical Services, Inc, North Little Rock, AR 72116, USA; 6 Abbott Laboratories, Abbott Park, Illinois 60064, USA

**Keywords:** Dog, Anesthesia, Clinical trial, Propofol

## Abstract

**Background:**

Propofol is a widely used injectable anesthetic agent for induction and short-term maintenance in dogs. A multi-dose formulation of propofol (MDP) has been developed which includes 2% benzyl alcohol as a preservative. In order to document the use of the product under clinical conditions, MDP was tested in a prospective clinical trial conducted at six sites within the United States. One hundred thirty-eight healthy, client-owned dogs were assigned to one of six treatment groups based on premedicants (none, acepromazine/buprenorphine, midazolam/buprenorphine, medetomidine/buprenorphine) and maintenance agents (MDP, inhaled anesthetic). Anesthesia was induced by the intravenous administration of MDP given to effect. Physiological indices including heart rate, respiratory rate and blood pressure were monitored prior to and during anesthesia induction, maintenance and recovery. Adverse events, defined for severity by pre-established limits of these physiological values, as well as side effects, defined as any observation outside the normal range, were noted.

**Results:**

The mean intubation dose was 7.6 ± 2.1 mg/kg for MDP alone and 4.7 ± 1.3, 4.0 ± 1.0 mg/kg and 3.2 ± 1.4 mg/kg when buprenorphine was used in combination with midazolam, acepromazine and medetomidine, respectively. Of the 32 adverse events, apnea (12 incidents), bradycardia (9 incidents) and hypotension (7 incidents) were most frequently recorded. Emesis, cyanosis and second degree heart block were each noted once and successfully resolved. The cause of a single death 2 days post-anesthesia was assessed as a surgical complication.

**Conclusions:**

MDP was found to be acceptable for use in healthy dogs for induction and short term maintenance of anesthesia when used alone and in combination with premedicants and inhaled anesthetics.

## Background

A formulation of propofol intended for one-time use in dogs has been approved in the United States since 1996 [1]. Since its introduction, propofol has gained widespread acceptance as an anesthesia induction and short-term maintenance agent in dogs. The drug has a rapid onset of action and when used for short-term maintenance, recovery times and quality are considered favorable relative to inhaled anesthetic agents [2].

These single-use injectable preparations of propofol available for veterinary use are oil-in-water emulsions containing no preservative [1,3]. Hence the label recommendation is that any drug that has not been used within six hours after the bottle or vial has been penetrated should be discarded to minimize the risk of contamination and possible adverse consequences to the patient. This can result in both drug waste and financial loss in many veterinary practice situations. A formulation allowing penetrated bottles to be stored and re-used for a period of time without significant risk of contamination would minimize these concerns.

One such alternative formulation of propofol has become available for intravenous use in dogs. The preservative in this formulation is 2% benzyl alcohol, a compound that has been used as a preservative in other injectable solutions (e.g., atropine) available for intravenous use. It has been shown to be highly effective against gram positive bacteria, molds and fungi, and moderately effective against gram negative organisms [4]. The addition of benzyl alcohol as a preservative to the original propofol formulation has resulted in a product that is labeled for a 28-day in-use shelf life [5]. Toxicity studies conducted under laboratory conditions on this formulation showed no significant adverse events when administered to dogs at up to 3 times the labeled induction dose [5].

The present study was designed to document the use of MDP containing 2% benzyl alcohol in dogs under clinical conditions.

## Methods

### Animals

One hundred and thirty eight client-owned dogs were enrolled in this study which was conducted at six sites, including three university veterinary hospitals and three privately owned veterinary clinics. Prior to inclusion a physical examination was performed on all dogs and results of a complete blood count and serum chemistry were evaluated. Animals that had laboratory values outside the normal range for a given facility, were pregnant, had received an investigational drug or had been enrolled in an investigational drug study within 30 days, or were categorized as ASA Category IV or V were excluded. Owner consent for participation was obtained prior to enrolling each animal in the study. Institutional animal care and use or hospital management committee approval was also obtained at Colorado State University, the University of Tennessee, the University of Florida and the private veterinary practices participating in the study. Once enrolled, demographic data including age, breed, gender and body weight were recorded.

### Study design

This study was designed to include at least 100 cases in which MDP was utilized as the induction agent prior to maintenance with MDP or an inhalant anesthetic. This number is considered adequate by regulatory agencies to determine safety for veterinary products under conditions of use. Dogs were assigned to one of six treatment groups based on premedicant combinations and maintenance agents they were to receive (Table 1). Group assignments were not randomized but rather were left to the discretion of the investigator who evaluated the patient’s physical status and laboratory findings and considered the nature and duration of the procedure to be performed. The use of anticholinergics during any phase of anesthetic management was also permitted at the discretion of the on-site investigator and injectable carprofen was allowed post-operatively.

**Table 1:** Group classifications and duration of procedures

**Group**	**Preanesthetic medications**^**1**^	**Maintenance agent**	**Number of dogs per group**	**Duration of procedure (min) Mean ± SD and range**^**2**^
**1**	None	Multidose propofol	23	14.6 ± 15.8
				0.3-62
**2**	Midazolam/buprenorphine	Multidose propofol	25	19.7 ± 14.6
				2-64
**3**	Acepromazine/buprenorphine	Multidose propofol	25	18.0 ± 14.3
				5-56
**4**	Midazolam/buprenorphine	Inhalant	22	41.1 ± 18.4
				10-75
**5**	Acepromazine/buprenorphine	Inhalant	22	73.6 ± 53.1
				20-203
**6**	Medetomidine/buprenorphine	Inhalant	21	38.3 ± 20.4
				7-98

^1^Anticholinergics were considered optional for all treatment groups. Anesthesia in all dogs was induced with multi-dose propofol. See text for additional detail.

^2^Statistically significant (p < 0.05) differences between treatment group means for duration of procedure: 4 > 1 and 5 > 1, 2, 3, 4, 6.

Due to the necessity of determining an appropriate anesthesia protocol for each dog, the study was not blinded. In addition to choice of the treatment group to which a patient was assigned, the on-site investigator was free to choose the dose and route of drugs used for premedication in each dog within broadly established guidelines designed to be in keeping with the wide range of dosages used in clinical practice. This information was recorded for all animals.

In animals receiving premedicants, anesthesia induction with MDP was initiated after the full effect of premedicants was present; MDP was delivered over a period of 60 – 90 seconds via an intravenous (IV) catheter until endotracheal intubation could be achieved. The start time and dose of propofol required for anesthesia induction and duration over which the dose was administered were recorded. To permit evaluation of the safety and efficacy of MDP in the face of repeated withdrawals, each vial was numerically identified and the duration of individual vial usage and the number of withdrawals per vial were noted.

Anesthesia was maintained with successive intravenous injections of MDP (groups 1–3) or with either isoflurane or sevoflurane (groups 4–6) in oxygen administered using a semi-closed rebreathing or non-rebreathing (Bain) circuit and out of circle vaporizer based on animal size and individual practice standards. Animals were allowed to breathe spontaneously. Maintenance agents (MDP or inhaled anesthetic) were administered to maintain an adequate plane of anesthesia for the procedure being performed; the dose and time of additional MDP administration was noted and the vaporizer setting (for the inhaled agent) was recorded at fixed intervals. The use of propofol to supplement inhalation anesthesia in the event of a sudden lightening of the anesthetic plane was permitted and recorded. In an effort to allow for the variability in clinical practice, oxygen supplementation was optional for dogs in the propofol maintenance groups.

### Behavioral and physiologic variables

The quality of induction and time to intubation from start of MDP administration were recorded. Times to extubation, sternal recumbency and standing following the last dose of MDP or from discontinuation of inhalation agent administration were also noted for all animals. Investigators assigned subjective scores reflecting the quality of induction, maintenance and recovery (Excellent, Good, Fair, Poor) based on previously published scales [6]. For example, an excellent induction would include a smooth rapid transition to recumbency without vocalization and muscle movement, whereas a poor induction would include muscle fasciculation, urination or defecation and inappropriate vocalization. The duration and type of intervention or surgical procedure were also noted.

Total anesthesia duration was considered from the time of intubation to the conclusion of drug administration (last dose of MDP for groups 1 – 3 and vaporizer off for inhaled anesthesia groups 4–6). The duration of drug induced recumbency following the induction dose of MDP was calculated for those animals maintained on propofol (groups 1–3) using the time from intubation to the time that the first additional (maintenance) dose was administered.

Rectal temperature (T), heart rate (HR), respiratory rate (RR), mucus membrane color, indirect systolic (S), diastolic (D) and mean (M) arterial blood pressure (BP) were recorded prior to premedication and again prior to anesthesia induction. Heart rate was either obtained by palpation of the pulse or recorded from an oscillometric non-invasive BP monitor used to obtain S, D and M arterial pressures prior to and during anesthesia; cuff size approximated 40% of the circumference of the limb and was placed proximal to the carpus or hock. Electrocardiographic (ECG) monitoring was not required for the study, but was performed in some animals at the discretion of the investigator. Hemoglobin saturation with oxygen (as measured by a pulse oximeter, SpO_2_) and end-tidal carbon dioxide (as measured by a capnograph, ETCO_2_) were monitored continuously and recorded immediately following intubation and again at 5, 10, 20, 30 minutes after intubation and then at 30 minute intervals during anesthesia maintenance. Respiratory rate was measured by observation of the chest wall or recorded from the capnograph. A thermistor or thermometer was placed in the esophagus or rectum, respectively, to record body temperature at the aforementioned time points during anesthesia maintenance.

Observations which included predetermined parameters of central nervous system activity (e.g., excitation, excessive depression/death), and limits for cardiovascular (e.g., hypotension, arrhythmias) and respiratory (e.g., tachypnea, apnea, hemoglobin oxygen saturation) system parameters, were recorded if observed prior to and at any time during anesthesia and recovery until the patient reached sternal recumbency. Observations of physiological values outside the normal range were considered side effects. Adverse events were defined as extremes in values or prolonged duration for these observations: apnea for greater than 120 seconds, a heart rate less than 50 beats per minute, mean arterial pressure less than 50 mm Hg, any abnormal ECG rhythm which upon evaluation was considered by the site investigator to be harmful to the patient, hemoglobin saturation with oxygen of less than 80% (for any duration) or between 80 and 90% for more than 3 minutes. Additional side effects such as excessive salivation and emesis were also noted.

### Statistical analysis

Differences among the six treatment groups in patient characteristics were statistically evaluated via either chi-square analysis (gender and ASA status) or one-factor analysis of variance followed by Tukey’s procedure for pair-wise mean comparisons (age and weight).

Data on each quantitative response variable (e.g., time to sternal recumbency and HR at times both prior to and following induction) were statistically summarized (mean and standard deviation) and were analyzed via analysis of variance (ANOVA) with treatment group as a fixed effect and also study site as a random effect. Because both the variation among sites and the interaction between sites and treatment groups were found to be uniformly non-significant (p > 0.10), the final ANOVA model included only treatment group as a fixed effect. The timed recovery variables (e.g., time to sternal recumbency) required a logarithmic transformation to satisfy the ANOVA assumption of Normality, which was evaluated via the Shapiro-Wilk test. Pair-wise comparisons of treatment group means were based on the Tukey-Kramer adjustment of probability-values for multiple testing. A p value of ≤ 0.05 was considered significant.

## Results

Because both the variation among sites and the interaction between sites and treatment groups in the ANOVA of each quantitative variable that included study site as a random effect were found to be uniformly non-significant (p > 0.10) the results presented here are those from the ANOVA which included only treatment group.

Patient demographics (age, weight, gender) and ASA status are presented in Table 2. Briefly, patients ranged in age from 0.25 to 17 years and represented multiple breeds. Both genders were well represented. The majority of patients were classified as ASA I and the remainder as ASA II. Among the breeds included in the study, there were 34 mixed breed, 13 Labrador Retrievers, 7 Yorkshire Terriers, 5 each of King Charles Spaniel, Golden Retriever, Jack Russell Terrier and Miniature or Toy Poodle, and 34 other breeds which were represented by 4 or less individuals. No significant differences in patient numbers, ASA status or weight were identified across groups. The mean age of group 2, in which mass removals were common, was greater than those of groups 4 and 5, in which spays and neuters were common. Also, differences in gender upon enrollment were observed between groups; there were statistically significant differences in number of intact versus spayed/neutered animals, but not males versus females. No statistically significant differences in baseline T, HR, BP or RR were identified across groups except for a difference in baseline RR between animals in group 5 (acepromazine/buprenorphine) and group 6 (medetomidine/buprenorphine) (Table 3).

**Table 2:** Patient demographics by treatment group

**Group**	**1**	**2**	**3**	**4**	**5**	**6**	**Total**
N	23	25	25	22	22	21	138
Age (Years)^1^							
Mean	4.65	7.70	4.84	3.95	2.87	5.67	5.00
SD	3.97	3.42	4.16	3.88	2.67	5.24	4.17
Range	(0.8-14.0)	(0.8-13.5)	(0.3-12.0)	(0.3-12.5)	(0.3-10.0)	(0.3-17.0)	(0.3-17.0)
Weight (kg)^1^							
Mean	15.5	21.6	15.7	14.0	20.7	14.8	17.1
SD	10.6	14.5	13.4	9.6	10.5	9.9	11.8
Range	(2–31)	(3–52)	(2–46)	(3–32)	(5–41)	(2–33)	(2–52)
Gender (N [% Total])^2^							
Male	0	2 (8)	8 (32)	8 (36)	13 (59)	7 (33)	38 (28)
Male castrated	9 (39)	10 (40)	10 (40)	2 (9)	2 (9)	7 (33)	40 (29)
Female	3 (13)	0	1 (4)	4 (18)	5 (23)	2 (10)	15 (11)
Female spayed	11 (48)	13 (52)	6 (24)	8 (36)	2 (9)	5 (24)	45 (33)
ASA status (N [% Total])^2^							
I	18 (78)	16 (64)	19 (76)	16 (73)	19 (86)	13 (62)	101 (73)
II	5 (22)	9 (36)	6 (24)	6 (27)	3 (14)	8 (38)	37 (27)

^1^Mean, standard deviation (SD) and range for age and weight; significant differences among groups in mean age with group 2 > groups 4 and 5 (p < 0.05).

^2^ N refers to the number of animals in each category of gender and ASA status; significant differences among groups in gender (p < 0.01).

**Table 3:** Mean ± SD of physiological variable measurements by treatment group

**Variable**	**Group**	**Immediately prior to premedication**	**Immediately prior to induction**	**Immediately post-induction**	**Minutes post-induction**			
					**5 min**	**10 min**	**20 min**	**30 min**
Heart rate (beats per min) (N)^1^	1	NA (0)	117.2 ± 32.6 (22)	118.0 ± 27.1 (21)	123.7 ± 26.2 (20)	119.7 ± 28.7 (18)	112.9 ± 21.4 (10)	114.8 ± 18.5 (5)
	2	113.0 ± 31.0 (25)	112.8 ± 32.2 (25)	116.3 ± 36.2 (23)	110.0 ± 28.7 (21)	112.1 ± 25.4 (19)	101.8 ± 26.7 (17)	94.2 ± 18.5 (14)
	3	107.6 ± 28.9 (25)	96.9 ± 25.8 (25)	99.5 ± 34.1 (25)	101.0 ± 30.7 (23)	100.3 ± 26.3 (24)	103.4 ± 22.3 (16)	99.4 ± 19.1 (7)
	4	127.8 ± 31.3 (22)	135.9 ± 35.0 (22)	146.8 ± 28.2 (20)	146.8 ± 30.9 (19)	141.7 ± 27.4 (18)	126.7 ± 26.0 (18)	123.1 ± 20.1 (19)
	5	116.8 ± 18.9 (22)	145.1 ± 44.0 (22)	142.9 ± 37.3 (21)	158.1 ± 32.0 (20)	134.2 ± 25.2 (21)	124.2 ± 23.6 (21)	114.5 ± 23.4 (20)
	6	115.6 ± 33.5 (21)	88.4 ± 36.9 (21)	94.9 ± 27.4 (20)	104.1 ± 26.3 (19)	102.6 ± 22.3 (19)	103.1 ± 20.4 (19)	95.1 ± 18.8 (18)
	Statistical results^2^	None	4 > (3, 6); 5 > (2, 3, 6)	4 > (2, 3, 6); 5 > (3, 6)	4 > (2, 3, 6); 5 > (1–3, 6)	4 > (2, 3, 6); 5 > (3, 6)	4 > (2, 3, 6); 5 > (3, 6)	4 > (2, 3, 6); 5 > (3, 6)
Respiratory rate (breaths per min)	1	NA	41.6 ± 13.8	28.6 ± 24.0	31.2 ± 16.7	32.3 ± 26.6	22.6 ± 11.5	23.5 ± 9.8
	2	41.1 ± 13.7	36.4 ± 14.9	26.1 ± 15.5	27.1 ± 18.7	30.7 ± 19.9	32.1 ± 18.4	27.0 ± 15.2
	3	30.6 ± 13.7	30.3 ± 13.3	23.0 ± 15.2	24.6 ± 11.6	29.3 ± 11.6	27.8 ± 10.8	26.4 ± 15.3
	4	34.8 ± 12.0	34.4 ± 11.8	23.9 ± 18.8	25.7 ± 13.3	24.9 ± 19.9	23.3 ± 21.7	26.5 ± 16.9
	5	28.6 ± 6.2	29.9 ± 17.5	12.6 ± 9.8	17.4 ± 15.3	17.0 ± 11.6	14.0 ± 6.3	12.6 ± 8.6
	6	42.4 ± 17.5	30.7 ± 23.6	12.2 ± 4.5	13.8 ± 6.4	13.8 ± 7.1	14.7 ± 6.7	12.9 ± 5.9
	Statistical results^2^	6 > 5	None	1 > (5, 6)	(1, 2) > 6	(1, 2, 3) > 6	(2, 3) > 5; 2 > 6	(2, 3, 4) > 5; (2, 4) > 6
Systolic blood pressure (mmHg)	1	NA	148.0 ± 28.8	128.1 ± 19.9	128.2 ± 32.8	127.2 ± 21.5	115.7 ± 18.2	112.8 ± 14.8
	2	141.8 ± 21.3	135.0 ± 30.6	119.6 ± 24.7	113.2 ± 24.0	115.3 ± 28.0	111.1 ± 20.8	115.9 ± 24.4
	3	136.6 ± 23.4	126.2 ± 33.6	112.3 ± 21.0	108.8 ± 18.5	112.2 ± 18.7	106.9 ± 17.3	106.1 ± 15.5
	4	129.6 ± 23.1	137.4 ± 24.9	122.7 ± 21.3	108.8 ± 15.3	106.4 ± 17.3	105.6 ± 26.7	108.6 ± 31.8
	5	137.2 ± 22.3	134.5 ± 16.5	124.4 ± 15.2	112.1 ± 17.1	100.9 ± 11.1	99.8 ± 14.7	101.3 ± 13.7
	6	137.1 ± 26.6	128.9 ± 34.5	125.9 ± 40.1	116.2 ± 39.4	127.9 ± 52.1	124.5 ± 36.2	116.6 ± 26.3
	Statistical results^2^	None	None	None	None	(1, 6) > 5	6 > 5	None
Diastolic blood pressure (mmHg)	1	NA	91.5 ± 33.6	81.1 ± 21.1	79.7 ± 32.2	82.8 ± 22.6	69.4 ± 14.9	74.0 ± 23.8
	2	89.0 ± 18.4	86.8 ± 22.8	77.1 ± 27.5	71.2 ± 16.2	73.3 ± 19.0	71.8 ± 22.9	75.1 ± 21.8
	3	92.4 ± 26.1	76.9 ± 27.4	70.4 ± 24.0	63.5 ± 18.5	63.8 ± 15.4	57.7 ± 21.3	58.7 ± 15.5
	4	87.1 ± 25.8	90.6 ± 28.6	76.2 ± 17.2	62.9 ± 11.6	57.9 ± 15.4	57.6 ± 19.8	60.6 ± 21.6
	5	98.8 ± 18.3	89.0 ± 17.8	74.3 ± 19.0	61.3 ± 16.6	55.1 ± 4.6	53.9 ± 9.1	53.2 ± 8.6
	6	98.4 ± 25.0	91.4 ± 31.3	90.0 ± 33.6	76.8 ± 35.2	88.2 ± 44.4	79.2 ± 29.2	71.8 ± 20.5
	Statistical results^2^	None	None	None	None	1 > 5; 6 > (3, 4, 5)	6 > (3, 4, 5)	(2, 6) > 5
Mean blood pressure (mmHg)	1	NA	114.7 ± 27.1	100.2 ± 17.7	101.9 ± 31.7	99.9 ± 20.8	88.1 ± 14.8	90.0 ± 21.4
	2	108.9 ± 17.3	102.3 ± 21.0	95.3 ± 24.2	85.8 ± 16.4	90.4 ± 26.0	87.5 ± 21.2	90.4 ± 24.1
	3	107.8 ± 24.8	96.2 ± 27.9	86.3 ± 21.3	83.7 ± 16.4	85.5 ± 13.9	78.4 ± 20.7	81.1 ± 16.3
	4	103.7 ± 23.8	109.8 ± 25.2	94.4 ± 18.7	78.0 ± 13.6	77.8 ± 15.4	76.6 ± 19.1	77.8 ± 22.0
	5	113.1 ± 20.1	105.6 ± 14.3	92.0 ± 18.0	79.7 ± 17.4	72.4 ± 8.9	69.5 ± 9.5	70.3 ± 10.4
	6	115.1 ± 24.8	107.1 ± 31.5	105.1 ± 36.2	92.6 ± 33.4	105.6 ± 44.9	99.5 ± 30.5	90.9 ± 19.3
	Statistical results^2^	None	None	None	1 > (4, 5)	1 > 5; 6 > (4,5)	6 > (3, 4, 5)	(2, 6) > 5
Hb Ox Sat (%)	1	NA	NA	90.0 ± 9.9	94.3 ± 3.0	94.5 ± 2.7	95.9 ± 2.2	97.3 ± 1.7
	2	NA	NA	90.1 ± 6.7	91.8 ± 6.7	93.4 ± 3.5	94.8 ± 2.7	97.7 ± 2.4
	3	NA	NA	93.9 ± 4.3	94.0 ± 3.9	94.6 ± 2.7	94.8 ± 3.2	97.5 ± 2.4
	4	NA	NA	95.7 ± 2.4	95.8 ± 2.1	96.7 ± 2.0	96.8 ± 1.7	95.4 ± 1.8
	5	NA	NA	96.5 ± 1.9	97.6 ± 1.2	97.2 ± 1.5	97.2 ± 1.8	97.0 ± 1.7
	6	NA	NA	95.6 ± 3.6	96.0 ± 2.4	95.7 ± 2.6	96.5 ± 2.6	96.5 ± 2.6
	Statistical results^2^	-	-	(4, 5, 6) > (1, 2)	(4, 5, 6) > (1, 2)	(4, 5, 6) > 2; 5 > 3	5 > (2, 3)	None
End-tidal CO2 (%)	1	NA	NA	28.9 ± 7.9	31.7 ± 8.3	32.5 ± 7.9	36.3 ± 5.5	40.3 ± 9.2
	2	NA	NA	34.8 ± 12.7	34.1 ± 8.0	38.2 ± 9.2	38.0 ± 9.4	40.4 ± 12.2
	3	NA	NA	31.6 ± 9.9	35.9 ± 9.8	36.0 ± 7.2	37.7 ± 9.6	47.0 ± 9.6
	4	NA	NA	34.6 ± 12.2	37.2 ± 9.4	36.8 ± 9.9	36.6 ± 8.7	38.7 ± 5.7
	5	NA	NA	30.5 ± 7.9	33.7 ± 6.3	36.5 ± 5.9	37.4 ± 4.6	38.4 ± 4.4
	6	NA	NA	37.0 ± 9.2	41.4 ± 7.3	40.8 ± 9.3	42.0 ± 8.0	43.8 ± 8.2
	Statistical results^1^	-	-	None	6 > 1	6 > 1	None	None
Rectal temp. (deg. F)	1	NA	101.7 ± 0.7	101.2 ± 0.8	100.9 ± 0.9	100.4 ± 0.9	100.1 ± 1.2	99.8 ± 1.0
	2	101.4 ± 0.7	101.3 ± 0.7	100.8 ± 0.8	100.4 ± 0.7	100.1 ± 0.7	99.8 ± 0.8	99.5 ± 0.8
	3	101.3 ± 0.8	100.9 ± 0.7	100.3 ± 0.8	100.0 ± 1.0	99.8 ± 1.3	99.5 ± 1.3	99.1 ± 1.6
	4	101.5 ± 0.8	101.2 ± 0.7	100.3 ± 1.3	100.3 ± 0.8	100.2 ± 0.8	99.6 ± 0.9	99.0 ± 1.0
	5	101.7 ± 1.0	101.3 ± 0.8	100.6 ± 0.9	100.4 ± 0.9	100.3 ± 0.7	99.9 ± 0.9	99.2 ± 1.1
	6	101.9 ± 0.6	101.5 ± 0.9	100.7 ± 1.3	100.5 ± 1.2	100.5 ± 1.3	100.1 ± 1.6	100.0 ± 1.5
	Statistical results^1^	None	1 > 3	1 > 3	1 > 3	None	None	None

^1^Number of animals for physiological variables measured at a given time point.

^2^Includes all statistically significant (p < 0.05) differences between means for treatment groups at a given measurement time point.

Procedures that dogs underwent during this study included castration (35), dental prophylaxis and/or tooth extraction (33), mass removal (19), radiographs (13), surgical ovariohysterectomy (laparotomy) (10), arthrocentesis, ear flush, grooming/nail trim (3), gastroscopy, auricular hematoma repair, cutaneous biopsy, joint injections, mass/skin tag removal (2) and adipose collection, arthroscopy/TPLO plate removal, endoscopy, enterotomy, entropion repair, epidural stem cell injection, gastrotomy, laparoscopic ovariohysterectomy, and allergy testing, shock wave therapy, skin testing and splint removal (1). The duration of procedures (average times per group listed in Table 1) ranged from less than one minute to 203 minutes.

The range of premedication doses administered to individual animals across all groups and study sites were: acepromazine (0.01-0.63 mg/kg [0.005-0.29 mg/lb] SC, IV or IM); atropine sulfate (0.02-0.075 mg/kg [0.009-0.03 mg/lb] SC or IM); buprenorphine (0.01-0.033 mg/kg [0.0045-0.015 mg/lb] SC or IM); medetomidine (0.004-0.028 mg/kg [0.002-0.013 mg/lb] IV or IM) and midazolam hydrochloride (0.02-0.48 mg/kg [ 0.009-0.22 mg/lb] SC, IV or IM). Based on pair-wise mean comparisons following ANOVA, the dose of acepromazine received in dogs in the MDP maintenance group was found to be significantly higher than for dogs maintained with inhaled anesthetics. The mean doses of midazolam and buprenorphine did not differ significantly between groups assigned to receive these drugs.

Propofol administration for anesthesia induction began an average of 27.6 minutes after premedication. The mean dose of MDP grouped by sedative or tranquilizer used is shown in Table 4. Dogs receiving no premedication received a significantly higher mean propofol dose (7.6 ± 2.1 mg/kg [3.4 ± 1.0 mg/lb]) than all other groups. Dogs receiving medetomidine for premedication received the lowest mean propofol dose (3.2 ± 1.4 mg/kg [1.4 ±0.6 mg/lb]) for anesthesia induction which was also significantly less than that for dogs receiving midazolam (4.7 ± 1.3 mg/kg [2.1 ± 0.6 mg/lb]), but not acepromazine (4.0 ±1.0 mg/kg [1.8 ± 0.5 mg/lb]). Actual administration time until the anesthetic endpoint for intubation ranged from 30 to 145 seconds resulting in rates of injection between 0.9 to 8.0 mg/kg/min (0.4 to 3.6 mg/lb/min). Investigators rated the quality of induction as excellent in 82 (59%), good in 47 (34%), fair in 8 (6%) and poor in 1 (<1%) of the 138 animals included in this study. Four of the nine inductions judged fair or poor involved dogs that were premedicated with midazolam/buprenorphine and in whom additional propofol was required for anesthesia induction. In groups maintained with propofol, the mean duration of anesthesia after the initial or induction dose (until subsequent dosing) was 5.8, 5.5 and 6.5 minutes in groups 1, 2 and 3, respectively.

**Table 4:** **Mean total multi-dose propofol (MDP) required for induction of anesthesia, grouped by premedicant**^**1**^

**Premedicant**	**None**	**Midazolam**	**Acepromazine**	**Medetomidine**
**Number of animals**	23	47	45	20
**Total MDP dose (mg/kg)**^**2**^	7.55	4.70	4.00	3.15
**Standard deviation**	2.14	1.34	0.95	1.38
**Range**	4.6-16.0	2.2-8.3	1.8-5.1	1.7-7.5

^1^Sedative and tranquilizers given in combination with buprenorphine; use of anticholinergics was optional. See text for additional detail.

^2^Statistically significant (p < 0.05) differences: None > (Midazolam, Acepromazine and Medetomidine) and Midazolam > Medetomidine.

The duration of anesthesia after the administration of each individual maintenance dose of MDP ranged from 1.7 to 20 minutes in unpremedicated animals (group 1) and 1 to 12.5 minutes in both groups of premedicated animals (groups 2 and 3). The total maintenance time (intubation to last administered dose) for groups 1–3 ranged from 1.7 to 37 minutes. The average additional propofol administered for maintenance of anesthesia in group 1 dogs was 3.2 ± 1.7 mg/kg (1.5 ± 0.8 mg/lb). Group 2 and 3 dogs required 1.7 ± 1.1 mg/kg (0.8 ± 0.5 mg/lb) and 2.0 ± 1.2 mg/kg (0.9 ± 0.5 mg/lb), respectively. This averaged to a mean maintenance dose rate of 0.48 mg/kg/min (0.22 mg/lb/min) in group 1 (non-premedicated animals) and 0.31 mg/kg/min (0.14 mg/lb/min) and 0.4 mg/kg/min (0.18 mg/lb/min) in group 2 (midazolam/buprenorphine) and group 3 (acepromazine/buprenorphine), respectively. These results were not significantly different. The total maintenance doses in groups 1, 2 and 3 ranged from 0 (no additional maintenance doses administered) to 24.7 mg/kg MDP. The average total doses (ranges) of MDP (induction plus maintenance), administered to groups 1, 2 and 3 were 10.1 (5.1-30.0), 9.3 (3.9-23.8) and 8.0 (2.5-28.7) mg/kg, respectively.

Of the two inhaled anesthetics used during the course of the study, isoflurane use accounted for 85.5% of the cases maintained by inhalant. Total maintenance time with inhalant anesthetics (groups 4–6) ranged from 16.2 to 245 minutes. Initial mean vaporizer settings were 2.1% for isoflurane and 3.7% for sevoflurane. Mid-maintenance vaporizer settings were 1.8% for isoflurane and 3.5% for sevoflurane. Nine animals received supplemental propofol during inhaled anesthetic maintenance to enhance the quality of anesthesia. Apnea occurred in one animal in conjunction with the administration of a supplemental dose of MDP.

Quality of maintenance with propofol alone was rated as good or excellent in 86% of patients overall; 100% of patients in group 1 and 68 and 92% of patients in groups 2 and 3, respectively. In group 2, quality of maintenance in 24% of the animals was rated as fair and 8% rated as poor. Quality of maintenance was rated fair in a single group 3 animal (4%) and was unrecorded in another one (4%).

Mean (±SD) recovery times are presented in Table 5. No statistically significant differences were noted in time to extubation, and only sporadic differences between treatment groups were noted for times to sternal recumbency (group 2 vs. group 4) and to standing (groups 2 and 5 vs. group 1). Investigators evaluated the quality of recovery as good to excellent in 91, 76 and 92% of animals in groups 1, 2 and 3, respectively, with the remaining animals receiving a rating of fair. In dogs recovering from inhalation anesthesia, the quality of recovery was rated as good to excellent in 82, 81 and 81% of the animals in groups 4, 5 and 6, respectively. Eleven percent of animals in these groups received a rating of fair, and 5% (three animals) were rated as poor.

**Table 5:** **Geometric mean recovery times for dogs by treatment group**^**1**^

**Group**	**Time to extubation**^**2 **^**(min)**	**Time to sternal recumbency**^**2 **^**(min)**	**Time to standing**^**2 **^**(min)**
	**Geometric mean (GSD)/range**		
1	8.0 (1.82) 2 – 41	9.7 (1.67) 3 – 44	14.6 (1.54) 8 – 56
2	11.1 (1.76) 5 – 41	15.2 (1.71) 6 – 45	24.4 (1.58) 10 – 48
3	10.1 (1.71) 4 – 25	13.3 (1.69) 5 – 29	18.7 (1.68) 7 – 48
4	7.3 (1.86) 3-36	8.8 (1.74) 4-38	15.2 (1.97) 4-55
5	9.0 (1.94) 3-33	12.4 (1.87) 3-41	24.0 (1.98) 5-115
6	8.0 (1.72) 4-22	10.6 (1.68) 4-25	18.1 (1.90) 6-105
Statistical results^2^	None	2 > 4	2, 5 > 1

^1^Time measured from last propofol injection (Groups 1–3) or vaporizer off (Groups 4–6). See text for additional details.

^2^Includes all statistically significant (p < 0.05) mean differences between treatment groups based on log-transformed data.

Mean values for physiological parameters prior to premedication and anesthesia induction and during the first 30 minutes of anesthesia are presented in Table 3. Differences between groups observed at each time point for measured parameters are also indicated in the table. Average HR was highest in groups 4 and 5. Significant differences were observed between groups 4 and 5 and groups 2, 3 and/or 6 at various time-points after premedication and between groups 5 and 1 at 5 minutes post-induction. Average RR tended to be higher in groups 1–4 compared to groups 5 and 6. Significant differences were observed between groups 1 and 6 immediately and up to 10 minutes post-induction, between groups 1 and 5 immediately post-induction and between groups 2, 3 and 4 compared to groups 5 and/or 6 at various time-points during inhalant maintenance. Average BP (S, D or M) tended to be highest in groups 1, 2 and 6 with significant differences between groups 3, 4 and/or 5 observed during the maintenance period. Although not statistically significant overall, a decrease in SBP was observed in 56% of the animals immediately post-induction.

Average SpO_2_ tended to be highest in groups maintained on inhalant, with results in groups 4, 5 and 6 significantly greater than those in groups 1 and 2 immediately and at 5 minutes post-induction and greater than those in group 2 at 10 minutes post-induction. The SpO_2_ was also significantly greater in group 5 than in groups 2 and 3 at various time-points during maintenance anesthesia. There were 25 animals in which SpO_2_ <90% was reported, generally immediately post-induction. Most of these animals (23/25) were maintained on MDP (groups 1–3). Of the group 1–3 animals, apparent hemoglobin desaturation was exclusively observed in animals not receiving supplemental oxygen. In four of these animals, supplemental oxygen was provided, but in the remaining 19 dogs, the transient apparent hemoglobin desaturation resolved without treatment. The ETCO_2_ tended to be highest in group 6 and was significantly greater than group 1 immediately and at 5 minutes post-induction. Rectal temperature was significantly greater in group 1 compared to group 3 prior to induction and immediately and at 5 minutes post-induction.

Table 6 summarizes clinical side effects and adverse events by group. The most frequently observed side effect was hypotension which generally occurred during inhalation anesthesia. In 12 of the 16 affected animals, additional intravenous fluids and/or ephedrine was used to treat low blood pressure. Apnea, the second most common clinical side effect observed, typically occurred within the first five minutes after induction. Apnea lasting 120 seconds occurred more commonly in group 2, usually when animals received a midazolam dose of 0.3 mg/kg or greater. As a result of prolonged apnea, five animals in this group received supplemental oxygen. Bradycardia occurred mainly during anesthesia maintenance and was seen most frequently in animals premedicated with medetomidine (n = 6), two of which had also received atropine premedication. Atropine or glycopyrrolate was used in four instances to treat low heart rate. Tachycardia was observed in two animals, both in the presence of atropine. In one of the dogs, a heart rate of 177 was observed immediately after premedication with acepromazine and atropine. No increase in heart rate was observed following injection of MDP, however, the heart rate increased to 253 bpm at the 0–5 minute interval, returning to 128 bpm at 5–10 minutes. In the second animal, (group 4) no increase in heart rate was observed immediately after injection of MDP, but increases up to 167 bpm were observed throughout the 30 minutes of inhaled anesthetic maintenance. Hypertension was recorded in three group 6 animals in which MAP ranged from 142–215 mmHg; all three animals had received atropine. Pain on injection of MDP was not observed in this study.

**Table 6:** Number of animals per group with side effects* or adverse events** recorded during the study

	**Treatment group**						**Total**
	**1**	**2**	**3**	**4**	**5**	**6**	
Number of patients in group	23	25	25	22	22	21	138
Number of patients receiving atropine	0	0	1	10	21	6	38
Side effect (Adverse event)	No. patients experiencing side effect or adverse event ( )						
Hypotension		1 (1)	1 (1)	3 (2)	9 (1)	2 (2)	16 (7)
Apnea	1 (2)	6 (6)	2 (1)	3 (2)	3 (1)		15 (12)
Bradycardia	1 (1)	1 (1)	2 (1)	1	1	6 (6)	11 (9)
Excitation	1	2	1	6	2	1	13
Tachypnea	1	2		3	1		7
Arrhythmias				1 (1)	1		2 (1)
Paddling	2	1				1	4
Fasciculation		2		1		1	4
Tenseness	1	1	1				3
Hypertension						3	3
Cyanosis	1				1 (1)		2 (1)
Tachycardia				1	1		2
Salivation	1					1	2
Emesis					1 (1)		1 (1)
Death					(1)		1 (1)

*Quantitative side effects defined as physiological measurements outside the normal range.

**Quantitative adverse reactions defined as: heart rate less than 50 bpm, apnea >120 seconds, mean arterial blood pressure < 50 mmHg, any potentially lethal ECG rhythm, mean O_2_ saturation <80% (any duration) or 90% for > 3 min.

There was one death associated with an animal included in the study. This occurred 2 days post ovariohysterectomy in a 6-year-old female Jack Russell Terrier assigned to group 5. A post-mortem examination was performed, and the cause of death was attributed to septic peritonitis from an ovarian pedicle abscess consistent with introduction of a foreign material at the time of surgery. Three other dogs were treated from the same vial of MDP without incident; one was treated before and two after the dog experiencing the adverse event.

The maximum duration of use for any individual vial used during the course of the study was 17 days, and the maximum number of withdrawals (needle penetrations) was nine. Approximately 50% of the vials were used over at least two days. On average, the vials remained in use for two days during which three withdrawals were made. Investigators did not observe a change in dosing or reduction in quality of maintenance for those animals who were dosed at the end of the use interval for any vial.

## Discussion

Prior multicenter clinical studies have confirmed propofol as a fast-acting anesthetic agent, with a favorable recovery and safety profile [1,3]. The single-use formulations of this drug, however, do not allow multiple withdrawals for more than six hours from first penetration which can result in wastage of the unused drug. The purpose of this study was to demonstrate the safety and efficacy of MDP under a wide range of clinical circumstances. Results of this study suggest that the addition of benzyl alcohol to the formulation of the sterile injectable has the potential to reduce wastage by allowing a shelf-life of up to 28 days after the initial withdrawal/needle penetration without altering the safety or efficacy of the preparation. While direct comparisons cannot be made in the absence of control groups in which preservative-free propofol was used, results of dosing and prevalence of side effects can be compared to those from published studies on single-use propofol.

The population in this study consisted of healthy individuals with an average age of 5 years; the youngest dog was 3 months old. The use of benzyl alcohol as a preservative in human parenteral solutions has been associated with a rare but fatal neonatal gasping syndrome in premature human infants and some immature animals [7,8]. Exposure associated with toxicities was at or above 99 mg/kg/day, a level well above exposure encountered with the use of MDP for induction and short term maintenance of anesthesia in this study. However given the exclusion of dogs less than 10 weeks of age, we cannot make any recommendations regarding use for this population of patients. Similarly, due to the use of MDP for only a short duration in this study (up to 37 minutes) we cannot comment on the safety of MDP for prolonged infusion as has been previously described for preservative-free propofol [9].

The induction doses in this study for unpremedicated animals were slightly higher than reported for single-use propofol [1,3]. The reasons for this difference are likely multifactorial. For example, population differences and factors related to the study, such as stimulating animals to record physiological parameters prior to induction, may have played a role. In addition, the rate of propofol administration may have influenced the total dose. For this study, the recommended rate of administration was 60 and 90 seconds or until the patient could be intubated. This was suggested in an effort to minimize any cardiopulmonary depression as is common after a rapid intravenous bolus of a fixed dose. Interestingly, prior reports suggest that slow administration can decrease [10,11] or increase [12,13] the amount of propofol used to achieve a fixed endpoint. It is thought that, while a slower rate allows for more gradual equilibration of the blood and brain concentrations, anesthesia may not be achieved if this is excessively prolonged [10,13]. Induction doses of 6.5 and 5.5 mg/kg are reported with administration of single-use propofol over 60–90 versus 10–30 seconds [1,3]; the results in this study are similar to the study in which the slower injection rate was used.

Unlike results in unpremedicated animals, the range of mean doses to achieve induction in premedicated animals were similar to that previously reported for single-use propofol [1-3,12-17]. In the current study, the presence of premedication reduced the propofol induction dose by 38, 47 and 58% for midazolam/buprenorphine, acepromazine/buprenorphine and medetomidine/buprenorphine combinations, respectively. A greater dose-sparing potency of medetomidine compared to that of midazolam or acepromazine has been reported for single-use propofol [18-21].

The mean duration of anesthesia for MDP (recorded from end administration to requirement for first additional dose) was comparable to that previously reported for single-use propofol for both unpremedicated and premedicated animals [1-3,13]. Similar to results reported for the single-use propofol [12,15], the dose required to maintain anesthesia remained consistent over time, suggesting that the MDP did not accumulate upon repeated administration over a short period of time.

Differences in premedicant doses, the use of atropine and supplemental oxygen, surgical procedures, and monitoring methods among the study animals and sites likely contributed to variations in physiologic measurements, and may have influenced the ability to detect differences among the treatment groups. Overall, however, the effects of MDP on physiological variables mirrored those seen in prior reports including multicenter clinical trials with single-use preparations [1,3,18,22-25]. The most common findings in all of these studies include a decrease in blood pressure and respiratory rate after propofol administration with minimal to no effects on heart rate beyond that observed with premedication. This suggests that the addition of benzyl alcohol to the formulation did not have any significant additional cardiovascular or respiratory effects. Tachycardia was reported in only two dogs in this study, both premedicated with atropine, which is likely to have played a role. The low incidence of tachycardia in this study differs from results observed in a tolerance study of MDP in which increases in heart rate were observed immediately after induction with MDP. The phenomenon appeared to be dose-related, with a maximum heart rate of 230 beats per minute seen at a dose of 19.5 mg/kg with little effect observed at 6.5 mg/kg [5]. It is unlikely that a dog would require a bolus dose of this magnitude (19.5 mg/kg), under typical clinical conditions. Consistent with other reports, heart rate was lowest in medetomidine-treated groups [24].

In all but the medetomidine premedicated dogs (group 6), average S, D and M blood pressure tended to decrease following MDP induction. This has been attributed previously to propofol induced veno-dilation which decreases cardiac preload [25]. The vasoconstriction induced by medetomidine likely offset these effects [26,27]. Respiratory rate decreased numerically in all groups following induction of anesthesia but the mean decrease was not statistically significant for any group. Interestingly, ETCO_2_ tensions tended to remain within the normal range throughout the study. Blood gas measurements would have helped determine the degree to which the decrease in RR may have contributed to changes in ventilation in individual animals but were not performed in this study. The average SpO_2_ immediately post-induction was 90.0 ± 9.9, 90.1 ± 6.7 and 93.9 ± 4.3 in groups 1, 2 and 3, respectively, the MDP maintenance groups (Table 3). This apparent hemoglobin desaturation was likely due to a combination of respiratory depression and drug or recumbency-induced ventilation perfusion abnormalities. Arterial blood gas analysis may have helped further elucidate the cause. Prior reports on a single-use preparation suggest that propofol-induced respiratory depression is exacerbated when sedating drugs and Mu (OP3) agonist opioids are used with propofol [21,28]. Approximately 37% of animals in the MDP maintenance groups (1–3) received supplemental oxygen prior to induction. Of those not receiving supplemental oxygen, the average SpO_2_ immediately post-induction was 85.3 ± 11.4, 86.2 ± 6.3 and 91.0 ± 5.2 in groups 1, 2 and 3, respectively. Although the low SpO_2_ was transient in dogs maintained on MDP, these data support the recommendation to provide supplemental oxygen to animals maintained on propofol [2]. The higher SpO_2_ observed in dogs maintained on inhalant anesthetics was expected, as these dogs were breathing a high fraction of inspired oxygen. This was similar to dogs in the MDP maintenance groups which received supplemental oxygen (average SpO2 immediately post-induction 95.1 ± 1.4, 94.8 ± 2.8 and 95.8 ± 1.9 in groups 1, 2 and 3, respectively).

The side effects observed were qualitatively comparable to those seen with the single-use product [1,3,5,13-17,19] and were predominantly related to the cardiovascular and respiratory systems. Apnea was more commonly seen during maintenance with propofol than inhaled anesthesia, but the incidence was lower than or comparable to that reported previously for the single-use product. The distribution of other cardiovascular effects between those animals administered propofol versus inhaled agents tended to mirror observations with single-use propofol, with the exception of hypotension, which was observed less frequently than expected in the MDP maintenance groups in this study. Other observed side effects were in keeping with those associated with specific premedicants. For example, poor sedation [29,30] and excitement has been reported as a side effect of midazolam [31], and hypertension has been associated with medetomidine [26,27] and the combination of medetomidine and atropine [29]. Abnormal ECG rhythms were observed only during inhaled anesthetic maintenance specifically in groups 4 and 5 (with 1 animal in each group). However, it was more common for dogs in these groups to be monitored in this manner. No ECG abnormalities were observed in a tolerance study of MDP at doses up to 19.5 mg/kg [5].

The toxicity of benzyl alcohol has been characterized in the dog [32] as well as other species [33-35]. Cardiovascular, respiratory and central nervous system effects, such as tremors, have been documented. Significant species differences have been noted, with the cat being particularly sensitive to benzyl alcohol toxicity, which is attributed to its glucuronide deficiency [36]. Parenteral toxicity of benzyl alcohol is also reported to be dependent on concentration in solution and rate of administration [32]. In the current study, the physiologic and clinical side effects of MDP were similar to those documented for single-use propofol. Also, side effects were not seen at higher levels in the groups maintained on MDP, despite their higher exposure. Thus, no specific adverse findings could be attributed to the addition of benzyl alcohol to the formulation. Despite the reported sensitivity of cats to benzyl alcohol, the anesthetic, physiologic and side effect profiles of MDP were also found to be similar in cats to those of single-use propofol [37].

There was no evidence of infection or sepsis as a result of MDP administration; the single case in which septic peritonitis was observed was attributed by the pathologist to the introduction of foreign material at the time of surgery. The use of the same vial of MDP in two dogs after the dog experiencing the adverse event precluded additional testing on this vial for contamination. However, laboratory testing of the MDP with repetitive needle punctures over the 28 days support the sterility of the product over the shelf-life [38].

The fact that approximately 50% of the vials were used over the course of at least two days, indicates that a preservative containing formulation can help eliminate waste from product remaining after the 6-hour recommended period for single-use (preservative-free) propofol.

## Conclusions

Based on the results of this study, MDP was found to be acceptable for use in ASA I and II dogs for induction and short term maintenance of anesthesia or induction of anesthesia prior to maintenance with inhaled anesthetics when used alone and in combination with commonly used premedicants. Induction and maintenance dose, duration of anesthesia after a single-use and anesthesia quality were comparable to those reported previously for single dose propofol. At clinically effective doses, no adverse effects could be directly linked to the addition of 2% benzyl alcohol used as a preservative in this formulation. The inclusion of benzyl alcohol in the formulation allowed the use of one propofol vial for up to 9 needle insertions for drug withdrawal over a period up to 17 days.

### Availability of supporting data

The data supporting this manuscript will not be included online.

## Abbreviations

ASA: American society of anesthesiologists; IM: Intramuscular; IV: Intravenous; MDP: Multidose propofol; SC: Subcutaneous.

## Competing interests

Elizabeth Cozzi is a stockholder and employee of Abbott Laboratories. Abbott Laboratories provided funding to Colorado State University, the University of Florida, the University of Tennessee, and the Animal Anesthesia and Pain Management Center for the conduct of the study and to Biotechnical Services, Inc. for the data management and analysis. MDP is a patented formulation that was developed by Abbott Laboratories. Abbott Laboratories provided funding to Khursheed Mama and Biotechnical Services for the preparation of the manuscript; there are no other competing interests.

## Authors’ contributions

EMC, KRM, JSG, SAR and RCH conceived and designed the experiments. EMC provided the test article (MDP). KRM, JSG, SAR and RCH performed the experiments. RLK and EMC summarized and analyzed the data. KRM, RLK and EMC prepared the manuscript. All authors read and approved the final manuscript.
